# Physical Exercise Restrains Cancer Progression through Muscle-Derived Factors

**DOI:** 10.3390/cancers14081892

**Published:** 2022-04-08

**Authors:** Argyro Papadopetraki, Maria Maridaki, Flora Zagouri, Meletios-Athanasios Dimopoulos, Michael Koutsilieris, Anastassios Philippou

**Affiliations:** 1 Department of Physiology, Medical School, National and Kapodistrian University of Athens, 11527 Athens, Greece; argpapa@med.uoa.gr (A.P.); mkoutsil@med.uoa.gr (M.K.); 2 Faculty of Physical Education and Sport Science, National and Kapodistrian University of Athens, 17237 Dafne, Greece; mmarida@phed.uoa.gr; 3 Department of Clinical Therapeutics, Alexandra Hospital, Medical School, National and Kapodistrian University of Athens, 11528 Athens, Greece; florazagouri@yahoo.co.uk (F.Z.); mdimop@med.uoa.gr (M.-A.D.)

**Keywords:** physical activity, exercise, muscle-derived factors, cancer, myokines, miRNAs, microRNAs, muscle-enriched miRNAs, exosomes, cancer progression

## Abstract

**Simple Summary:**

The benefits of physical exercise against cancer onset and progression, as well as the adverse effects of physical inactivity have changed the way that we utilize exercise for cancer patients. Nevertheless, although guidelines of various scientific societies and organizations propose exercise as a complementary intervention during cancer therapies, the exact cellular and molecular mechanisms by which exercise acts against cancer have not yet been elucidated. In the present review, we analyze the factors which either are secreted from skeletal muscle or are regulated by exercise and can restrain cancer evolution. We also describe the exercise-induced factors that counteract severe side effects of cancer treatment, as well as the ways that muscle-derived factors are delivered to the target cells.

**Abstract:**

A growing body of in vitro and in vivo studies suggests that physical activity offers important benefits against cancer, in terms of both prevention and treatment. However, the exact mechanisms implicated in the anticancer effects of exercise remain to be further elucidated. Muscle-secreted factors in response to contraction have been proposed to mediate the physical exercise-induced beneficial effects and be responsible for the inter-tissue communications. Specifically, myokines and microRNAs (miRNAs) constitute the most studied components of the skeletal muscle secretome that appear to affect the malignancy, either directly by possessing antioncogenic properties, or indirectly by mobilizing the antitumor immune responses. Moreover, some of these factors are capable of mitigating serious, disease-associated adverse effects that deteriorate patients’ quality of life and prognosis. The present review summarizes the myokines and miRNAs that may have potent anticancer properties and the expression of which is induced by physical exercise, while the mechanisms of secretion and intercellular transportation of these factors are also discussed.

## 1. Introduction

It is well established that the engagement in regular physical activity programs reduces the incidence of many non-communicable diseases, including cancer, and could be a powerful non-pharmaceutical therapy against the progression or recurrence of the disease [1,2,3,4,5]. Indeed, epidemiological studies have revealed that health-related lifestyle behaviors, such as high physical activity levels and healthy eating habits, could prevent 40% of all cancer cases in the United States, while, indicatively, regular exercise lowers the risk of developing breast cancer, the most frequently diagnosed female cancer, by about 30% to 40% [6,7].

Even though several mechanisms have been proposed to explain the benefits of physical exercise against cancer, the exact molecular mechanisms that mediate the exercise-induced antitumor effects are insufficiently understood [8]. Nevertheless, it has long been known that a single bout of exercise induces acute responses of physiological systems, and, consequently, accumulative bouts of exercise stimulate various systemic, tissue, and cellular adaptations including enhanced tumor-resistant inflammatory and immune responses, hormonal and metabolic alterations, angiogenesis, DNA repair and methylation, and expression of microRNAs (miRNAs), which “fight” against carcinogenesis [8,9,10,11].

**Figure 1:**
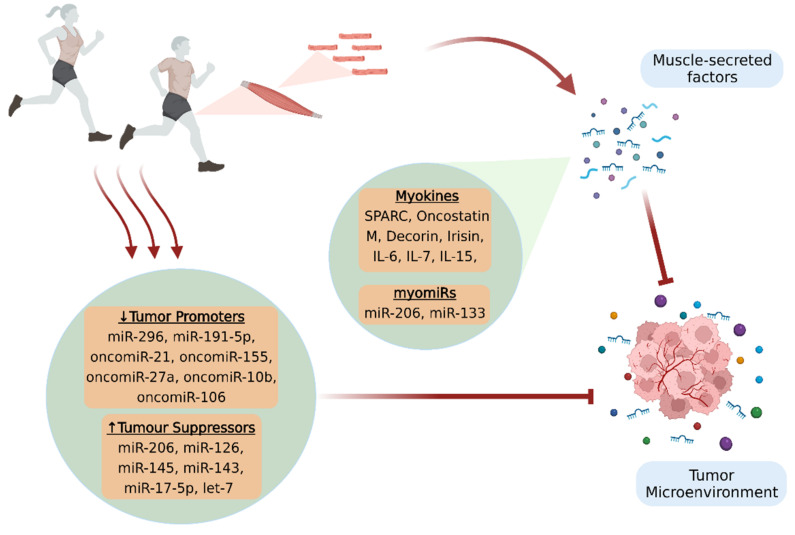
Physical exercise provides significant benefits against cancer, in terms of prevention and treatment, by affecting the TME. Skeletal muscle secretes bioactive molecules (myokines and muscle-enriched miRNAs, myomiRs) in the extracellular space through its contraction, which fight against tumorigenesis and disease progression. Moreover, exercise appears to promote beneficial expression patterns of circulating miRNAs, since it results in increased production of tumor suppressors and in the inactivation of tumor promoters, thus creating an unfavorable microenvironment for cancer progression. The figure was created with BioRender.com (accessed on 10 February 2022).

A determinant of all the above-mentioned processes that can modulate body homeostasis is skeletal muscle function. Indeed, skeletal muscle is the largest endocrine organ in the human body and plays an essential role in integrative physiology, as it has the ability to produce and release through its contraction a wide variety of biological molecules involved in inter-tissue cross talk [12]. Specifically, bioactive molecules produced by skeletal muscle and secreted into extracellular space are considered to be the myokines, growth factors, chemokines, exosomes, and skeletal muscle miRNAs. Current research evidence reveals that these muscle-derived factors influence directly or indirectly the tumor microenvironment (TME), demonstrating therapeutic potential [13]. Furthermore, there are biomolecules that although they are not secreted by skeletal muscle, their expression seems to be regulated by physical exercise [14] (Figure 1).

The present review focused on the exercise-regulated factors that possess potential anticancer properties and are involved in cancer progression and prognosis, with emphasis being given to the muscle-derived factors and the mechanisms of their secretion, intercellular transportation and translocation to the target cells.

## 2. Myokines

Over the last few decades, various secreted factors including cytokines, peptides, or growth factors have been identified as “myokines”, since they are produced and released by skeletal muscle cells under contraction [15,16,17]. Myokines do not only act locally within the muscle or the neighbor tissues but also affect distant organs, being the mediators of inter-tissue communication [18]. Thus, acting in an autocrine, paracrine, or endocrine manner, they form or activate molecular interactions and pathways between the contracting muscles and the entire body, exerting benefits against several diseases, e.g., those associated with chronic inflammation, oxidative stress, and metabolic disorders [9,19,20].

Moreover, a growing body of evidence reveals that myokines participate in the modification of the TME, contributing to both cancer prevention and evolution by exerting the beneficial effects of physical exercise and counteracting the harmful effects of cancer disease and the side effects of its treatments [21]. More specifically, myokines could be divided into those that may directly influence the TME, such as secreted protein rich in cysteine (SPARC), oncostatin M, and irisin; and those that may indirectly affect cancer evolution by enhancing the antitumor immune response, such as IL-6, IL-7, and IL-15 [22].

In this context, multiple in vitro studies in breast, lung, colon, or prostate cancer cell lines have shown that the treatment of cancer cells with exercise-conditioned human serum resulted in decreased metabolic activity and increased apoptotic rates in those cells [23,24,25,26]. Interestingly, however, the human post-exercise serum used for cell treatments did not affect the viability of normal cells but decreased the viability only of cancer cells [27,28]. These findings indicate that some of the biomolecules released into the bloodstream after exercise may possess the ability to decrease tumor cell growth and survival. In the following sections, secretory molecules that have been reported to potentially modulate tumor development and cancer-related phenotype are discussed.

### 2.1. Myokines and Cancer Progression

Given that adequate research data supporting a direct association between myokines and tumor growth are still lacking, SPARC is one of the most studied myokines in cancer [29]. SPARC, also known as osteonectin, is a matricellular protein implicated in the interactions of cells with the extracellular matrix (ECM) [30,31]. It has been found that SPARC is secreted from skeletal muscle into circulation after a single bout of exercise in healthy humans, but also in rodents with colon cancer. Moreover, it has been showed that regular exercise suppressed colon tumorigenesis in mice, while the anti-tumor effect of exercise was abolished in SPARC knockout mice [32,33]. These findings are in agreement with other studies that revealed increased SPARC expression in both physically active mice and humans, as well as a better overall survival in patients with digestive tract cancer who exhibited a higher than the median level of SPARC [34].

Furthermore, evidence from both in vitro and in vivo studies supports the notion that oncostatin M (OSM), a cytokine belonging to the IL-6 family [35,36], possibly mediates some of the inhibitory effects of exercise against cancer evolution. Indeed, it has been reported that the incubation of human breast cancer cells with a post-exercise human serum containing OSM inhibited cell proliferation and induced apoptosis, while the blockage of OSM mitigated the anti-tumor effects of exercise-conditioned serum [9]. The role of OSM as a myokine was further verified, as a single exercise bout resulted not only in the upregulation of OSM in skeletal muscles, but also in its increased secretion into the circulation [9]. Moreover, animal studies have confirmed that aerobic exercise exhibits its protective effects against cancer through OSM, resulting in decreased tumor volumes in tumor-bearing mice [37,38].

Decorin is a small extracellular matrix proteoglycan, which was recently identified as a myokine [39], as it has been reported to be secreted from skeletal muscles following exercise both in mice and humans [40]. In response to exercise stimulus, decorin acts to regulate muscle mass loss by inhibiting myostatin, another myokine that is widely known for its role in inducing muscle atrophy and sarcopenia [41] (discussed in the next section). Apart from the counteracting impact of decorin on muscle cachexia (discussed in the next section), its action has been associated with the restraint of tumor growth [42]. Specifically, it is considered that decorin suppresses the proliferation rates and metastatic potential of multiple types of cancer cells, such as hepatocellular carcinoma, breast cancer, and non-small-cell-lung cancer [40,41,43]. Various pathways have been proposed to mediate the beneficial effects of decorin against tumorigenesis. Part of its action is assumed to rely on the downregulation of transforming growth factor beta (TGF-β) that leads to the reduction of miR-21 or cyclin D1 [40] and to the increased expression of p53 and p21, which are major tumor-suppressing genes [43]. Moreover, in vitro studies in hepatoma cell lines indicated that the anti-tumor impact of decorin is probably associated with its interaction with surface cell receptors, like epidermal growth factor receptor (EGFR), insulin-like growth factor 1-receptor (IGF-1R), and other major tyrosine kinase receptors (TRKs). However, it has been also suggested that this myokine acts as a tumor suppressor by upregulating cell cycle-associated genes, such as p21WAF1/CIP1, p27KIP, and p57KIP2, and possibly by blocking the transition from G2 to M phase [44].

The myokine irisin, encoded by the *Fndc5* gene, is well known for its key role in the regulation of fat metabolism, favoring the switch from white to brown adipose tissue [45], and the stimulation of glucose uptake from skeletal muscles [46]. Recently, it has been observed that recombinant irisin significantly decreased the ability of breast cancer cells to proliferate and migrate, enhancing the cytotoxic activity of the common anti-neoplastic agent doxorubicin, without affecting the viability of non-malignant cells [47]. Other studies have also reported that irisin suppressed cell growth and induced cell cycle arrest in in vitro models of prostate cancer and glioblastoma [48,49]. Moreover, it has been observed that even a small increase in serum levels of irisin could limit the probability of developing breast cancer up to 90% [50], while higher levels of irisin in serum act in a protective manner against spinal metastasis in female breast cancer patients [51].

Ιt is noteworthy that Interleukin (IL)-6, the founding member of myokines, acts in a pleiotropic manner regarding cancer evolution, since different origins and signaling pathways have been associated with either its anti- or pro-tumorigenic properties [52]. Thus, the over-secretion of IL-6 in TME, not only by the tumor cells themselves [53] but also by cancer-associated macrophages [54], fibroblasts [53], adipocytes [55], or mesenchymal stem cells [56], creates favorable conditions for tumor development and metastasis. On the other hand, the release of IL-6 from skeletal myocytes in response to exercise indirectly inhibits neoplasm onset and development by increasing the mobilization and infiltration of cytotoxic immune cells, thus engaging muscle–immune cell crosstalk [57]. Specifically, Pedersen et al. showed that exercise regulates natural killer cell (NK) trafficking through epinephrine, and this beneficial effect is blunted with the blockage of IL-6, concluding that exercise contributes to the reduction of tumor growth rate by mobilizing IL-6 sensitive NKs [57]. Indeed, NKs belong to the fast-responding component of innate immunity, retain an important role in potentiating humoral and cellular adaptive responses, and are capable of eliminating multiple cell populations that have acquired a surface expression profile associated with oncogenesis [58].

In addition, other myokines of the interleukin family, such as IL-7 and IL-15, are indirectly implicated in exercise-induced anticancer immunity. Specifically, evidence supports that IL-15 regulates the proliferation and maintenance of NKs as well as the expansion of different T-cell subpopulations, including types of effector T-cells that actively mediate immunity (e.g., CD8+ cytotoxic T-cells, CD4+ T-helper cells, etc.), or cell types that contribute to immunological memory (such as CD8+ memory T-cells) [59,60]. Indeed, in the first IL-15-based clinical trial conducted on patients with metastatic malignancies, elevated levels of NKs and gamma delta (γδ) Τ-cells were detected in circulation upon administration of recombinant IL-15. Interestingly, a substantial clinical benefit was observed in two patients with melanoma who experienced complete lung metastases clearance [61]. Concerning IL-7, it seems to retain a key role in the homeostatic replenishing of T-lymphocyte pools by preserving survival and proliferation rates of young naive T-cells produced in the thymus [60,62]. Of equal importance is also the fact that IL-7 not only provides signals for the production of memory T-cells, but also sustains their presence during immune responses [63].

### 2.2. Myokines and Cancer-Associated Sarcopenia

Cancer-associated sarcopenia consists a severe muscle wasting syndrome manifesting in various cancer types, and it not only deteriorates patients’ functional ability and quality of life but can also lead to cancer death [64,65]. Sarcopenia may appear in cancer patients as a side effect of the systemic cytotoxic chemotherapies, or as a consequence of the tumor-secreted factors that disrupt skeletal muscle homeostasis and lead to increased proteolysis and suppressed protein synthesis [64]. In particular, selective atrophy of type 2 fibers with a fast-to-slow fiber type shift has been described in cachectic cancer patients [66]. In this context, physical exercise plays a pivotal role in maintaining skeletal muscle mass through the secretion of various myokines during muscle contraction [65].

Specifically, IL-6, whose anticarcinogenic properties have been already discussed, increases acutely after an exercise bout in both healthy subjects and cancer patients [67]. One of the essential muscle mass-related features of IL-6 is that it facilitates the proliferation, differentiation, and fusion of satellite cells by activating or regulating the respective JAK/STAT, p38/MAPK, and NF-κB signaling pathways. Thus, the involvement of IL-6 in satellite cell-dependent myogenesis can promote skeletal muscle protein synthesis and hypertrophy and ameliorate cancer-related muscle wasting [52,68].

Musclin, a recently characterized myokine encoded by the *Ostn* gene, has been shown not only to enhance aerobic capacity through the stimulation of mitochondrial biogenesis (via the regulation of peroxisome proliferator-activated receptor gamma coactivator 1-α (PGC1-a)), but also to be implicated in cancer-related muscle cachexia [69]. Indeed, low levels of musclin have been observed in atrophying skeletal myotubes and in plasma and muscles of cancer-bearing mice, while the electroporation of musclin-encoding plasmids nullified muscle atrophy manifestations in renal cancer-bearing mice [6,70].

Furthermore, stromal derived factor 1 (SDF1) is a newly introduced myokine encoded by the *CXCL12* gene, and its secretion from skeletal muscle is triggered by aerobic exercise [71]. SDF1 binds primarily to CXC chemokine receptor 4 (CXCR4), while the CXCL12/CXCR4 axis has a significant role in tumor progression, metastasis, and cancer cachexia [71,72]. In animal models of various types of cancer, such as hepatoma, colon adenocarcinoma, or renal cancer, SDF1 was found to be downregulated in muscles eliciting cancer-associated atrophy [73]. Additionally, SDF1 levels were negatively correlated with two well-known atrophy-related ubiquitin ligases, namely atrogin-1 and muscle RING-finger protein-1 (MuRF-1), in abdominal muscle of patients with cancer [73].

On the other hand, myostatin, a muscle-specific protein encoded by the *Mstn* gene in humans, is produced and released from skeletal muscle cells to inhibit their growth. Exercise decreases myostatin secretion and regulates the myostatin/ActRIIB pathway, which leads to protein degradation and muscle atrophy [74,75]. More specifically, myostatin binds to the membrane receptor ActRIIB and induces Smad phosphorylation, which may lead to the downregulation of Akt (protein kinase B), a key signaling protein for skeletal muscle hypertrophy, or the overexpression of the most common muscle atrophy genes (atrogenes), *atrogin-1* and *MuRF-1* [76]. In cachectic animal models with diverse malignancies, increased myostatin levels were related to muscle wasting, while the pharmacological abrogation of myostatin prevented muscle mass loss and prolonged survival [77,78,79,80]. In line with the findings of the animal studies, results of clinical trials administering anti-myostatin antibodies revealed increased muscle volume and lean mass in sarcopenic cancer patients without causing major side effects [81]. Similarly to myostatin, high levels of activin A (ActA) have been reported to cause muscle wasting in rodents and have been associated with cancer-related cachexia in humans [82]. The ActA protein is the major dimer of the activins, which belong to the TGF-β superfamily [78,83] and is primarily expressed in gonadal tissues [84]. Interestingly, however, it has been recently shown that it is also released from skeletal muscle cells and contributes to tumor-related muscle atrophy, mimicking the action of myostatin and sharing the same receptor (ActRIIB) [78,85,86].

Overall, the engagement in exercise training programs after cancer diagnosis represents a promising complementary therapeutic intervention that can prevent or alleviate muscle wasting by enhancing muscle anabolic processes and suppressing muscle protein catabolism.

## 3. Circulating microRNAs and MyomiRs

MiRNAs are a class of endogenous, single-stranded, non-coding RNAs with a length of 18–22 ribonucleotides [87,88,89,90,91]. MiRNAs cannot be translated into proteins, but rather they control post-transcriptional regulation of gene expression through cleavage, destabilization, or less efficient translation of coding mRNAs [92].

It has been well documented that the binding of miRNAs to the 3′-untranslated regions (3′-UTR) of their target genes alters their expression [88,91] and plays a vital role in the regulation of numerous physiological processes, including cell proliferation, differentiation, apoptosis, and metabolism [87,88,89,90]. Indeed, adequate evidence suggested that either the elevated or decreased levels of particular miRNAs are involved in a variety of human diseases, including cancer [90]. Specifically, the expression of specific miRNAs can lead to tumor suppression through the downregulation of oncogenes or the upregulation of tumor suppressing genes, while conversely the overexpression of other miRNAs, called oncomiRs, promotes oncogenesis [87,92]. For instance, miR-152 acts as a tumor suppressor in ovarian, gastric, and liver cancer, implicated in the inhibition of cell proliferation, invasion, and migration [93]. On the other hand, miR-24 has been identified as an oncomiR responsible for the bad prognosis of various types of non-solid and solid cancers, including leukemia and breast, liver, and lung cancer [94,95,96,97].

Even though the majority of miRNAs is expressed in numerous tissues, some of them are considered as tissue-specific, since they are transcribed as much as 20 times higher in specific cell types, compared with their expression levels in other tissues. In particular, myomiRs consist of a subcategory of miRNAs that are striated muscle-specific and are expressed in higher levels in skeletal and/or cardiac muscle [98].

Moreover, miRNAs are not detected exclusively in tissues and organs, but they can also be released into circulation (c-miRNAs), travel through the human body, and impact key cellular processes. Thus, while multiple c-miRNAs are associated with either carcinogenesis, tumor suppression, DNA repair, or checkpoint functions, they could also be potential mediators of the benefits that regular physical activity induces towards the regulation of cancer development and progression [92].

### 3.1. MyomiRs and Cancer Progression

MyomiR-133 is a circulating miRNA that not only influences myoblast differentiation but also contributes to the suppression of several tumors, such as ovarian, breast, prostate, gastric, bladder, pituitary, glioma, and colorectal cancer [99,100,101,102,103]. In this context, it has been shown that both acute and chronic exercise increases the intramuscular expression and the subsequent release of myomiR-133 into circulation, which subsequently impacts cancer progression by targeting crucial oncogenes, such as IGF-1R and EGFR [104,105,106]. These growth factor receptors interact with the PI3K/Akt and the MAPK/ERK signaling pathways, which orchestrate core cellular functions such as proliferation, differentiation, and apoptosis. Consequently, the upregulation of myo-miR-133 can abrogate cancer-associated hallmarks, such as aberrant cell migration and invasion, thus restraining cancer evolution [107].

Moreover, myomiR-206 is also a well-known tumor suppressor and inhibitor of cancer cell invasion [108,109,110], and it has been shown that when MCF-7 human breast cancer cells were transfected with miR-206, cell growth was restricted, and apoptosis was increased, while its expression is increased after aerobic exercise [111] (see next section).

### 3.2. MicroRNAs Regulated by Exercise

Recent evidence suggests that 45 min of aerobic exercise can acutely modify the expression of 14 c-miRNAs, which are involved in cancer pathways [111]. In particular, myomiR-206, a regulator of cancer cell proliferation and migration that plays an anti-cancer role in cancer progression [112,113], exhibited greater expression changes after aerobic exercise [111]. In addition, Isanejad et al. have shown that a 5-week interval aerobic exercise training program combined with hormonal therapy resulted in the upregulation of miR-206 and let-7 and in the downregulation of oncomiR-21 in breast cancer-bearing mice [114]. These findings are in line with previous studies showing that let-7 was elevated in healthy, regularly exercising women in contrast with sedentary ones [104], while decreased expression of c-miR-21 was found after regular exercise training in female mice with breast cancer [115]. Similarly to the actions of miR-206, let-7 is a tumor suppressor that targets the translation of many oncogenes, including *HRAS, KRAS, NRAS*, and *c-Myc* [104]. In contrast, c-miR-21 is a well-studied miRNA that promotes tumor growth and invasion through several pathways, such as the upregulation of the anti-apoptotic factor B-cell lymphoma 2 (Bcl-2) [116,117]. Moreover, c-miR-21 has been correlated with the overexpression of hypoxia-inducible factor 1-alpha (HIF-1α) and vascular endothelial growth factor (VEGF), two factors that predominantly determine the efficacy of angiogenesis towards oncogenesis, through vessel destruction and re-sprouting [114].

Cancer progression could be also influenced by the exercise-induced regulation of miR-296 and miR-126 expression in breast cancer. A 10-week aerobic exercise program in tumor-bearing mice led to decreased tumor growth mediated by the downregulation of the pro-angiogenic miR-296 and the upregulation of the anti-angiogenic miR-126 [118]. The decreased expression of VEGF-A and the overexpression of hepatocyte growth factor-regulated tyrosine kinase substrate (HGS) in the cancerous tissue confirmed that the angiogenetic pathway is involved in exercise-induced benefits against cancer progression [118]. Additionally, the increased expression of c-miR-126 in response to exercise could alter the TME and inhibit cancer cell invasion and metastasis by suppressing the expression of stromal cell-derived factor-1 alpha (CXCL12) and chemokine ligand 2 (CCL2) [92]. Indeed, cancer expansion is depended upon the capability of stromal cells to promote tumor invasiveness, give rise to new vessels, and suppress immune function [119].

Similarly, a randomized trial investigating the effect of 12-week high-intensity interval training in breast cancer patients under hormonal therapy revealed an altered expression pattern of many cancer-related miRNAs that initially exhibited an aberrant expression in these patients compared to healthy controls [120]. Specifically, the exercise intervention resulted in the downregulation of oncomiRs miR-21, miR-155, miR-27a, and miR-10b and the upregulation of the tumor suppressors miR-206, miR-145, miR-143, and let-7a [120].

Furthermore, interesting correlations were revealed when 221 women with breast cancer, enrolled either in the HOPE or LEAN trial [121,122], participated in supervised physical activity programs [123]. Specifically, from the eight miRNAs, namely miR-191-5p, miR-17-5p, miR-103a-3p, miR-93-5p, miR-22-3p, miR-122-5p, miR-126-3p, and miR-150-5p, that were identified in serum to be affected by body mass index (BMI; kg/m^2^), a strong positive correlation was found between BMI and miR-22-3p and miR-122-5p, which both are involved in the reprogramming of systemic metabolism, a predominant hallmark of cancer [124,125]. BMI was also negatively correlated with miR-191-5p and miR-17-5p expression levels, which have been associated with suppressed cancer cell invasion and metastasis in breast cancer [126,127,128]. Moreover, six miRNAs, namely miR-27a-3p, miR-191-5p, miR-24-3p, miR-106-5p, miR-92a-3p, and let-7b-5p, differed significantly in the women with breast cancer who completed the combined exercise and nutrition intervention as compared with the usual care group [123]. In particular, the carcinogenic miR-106-5p, which was significantly decreased in the intervention group, has been linked with poor prognosis and metastatic potency via the PI3K/Akt signaling pathway [129,130].

Overall, although there is not sufficient research evidence from human studies providing a direct association between physical activity and alterations in miRNA expression patterns in cancer patients, the afore-mentioned data indicate, however, that there is a mechanistic connection at the molecular level between physical activity, cancer risk, and cancer progression, since exercise results in acute responses and/or chronic adaptations of c-miRNA expression that influence cancer development.

## 4. Intercellular Transport and Delivery of Muscle-Secreted Biomolecules: The Role of Exosomes

Communication between different cell types and tissues is of vital importance both in health and disease, and skeletal muscle cells effectuate this process in a direct or indirect manner. Specifically, the bioactive molecules secreted by skeletal myocytes may act locally in a paracrine or autocrine manner, or they can be secreted into the circulation and travel and migrate through the body, acting in an endocrine manner. In general, autocrine, paracrine, and endocrine regulatory systems include active forms of secretion and transport of molecules that require energy expenditure, as well as the passive transport of substances across cell membranes without using cell energy [131].

Typically, in the framework of active intercellular communication, the formation of transport vesicles derived from the endoplasmic reticulum and subsequently from the Golgi apparatus is a common process for targeted substance trafficking [132]. These structures are called extracellular vesicles (EV) and enable the physiological translocation of molecules such as enzymes, cytokines, and miRNAs, which otherwise could not exit the cytosol and be released in the extracellular space or enter the circulation. In general, EVs are divided in three main categories according to their diameter: the exosomes (30 to 150 nm), the microvesicles (about 1 μm), and the apoptotic bodies (1 to 5 μm) [133,134]. The primary cellular process that mediates the exchange of bioactive molecules is exocytosis, which in cooperation with endocytosis, membrane fusion, and receptor–ligand binding, enables their uptake from target cells [135].

**Figure 2:**
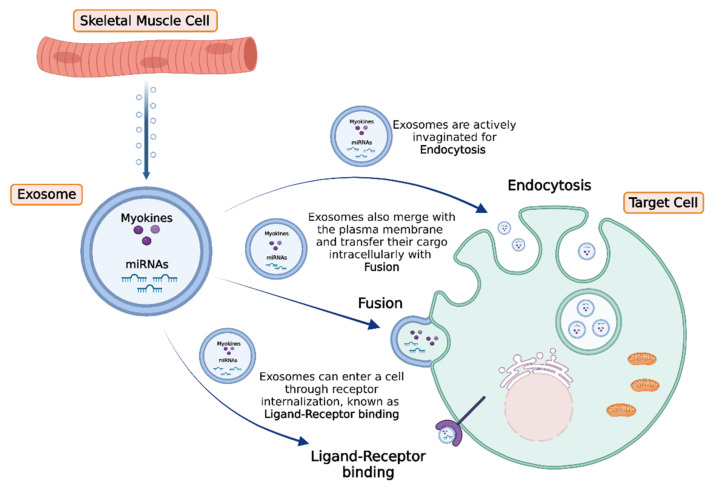
Exosomes are released from skeletal muscle cells in response to exercise stimuli, delivering their content (miRNAs and myokines) to target cells. Subsequently, target cells can uptake the exosomes by three main processes: (a) active endocytosis with invagination, (b) direct membrane fusion, or (c) internalization through ligand–receptor binding. The figure was created with BioRender.com (accessed on 10 February 2022).

Recently, it has been revealed that upon exercise stimuli, skeletal muscle cells release EVs to exert significant effects either to adjacent or distant tissues [42,136] (Figure 2). More specifically, myokines, along with other peptides, chemokines, and hormones, can be packed in specialized vesicles, the exosomes, the biogenesis of which requires the invagination of the plasma membrane to form an early endosome. Subsequently, the early endosome buds into the surrounding lumina, leading to the formation of many small intraluminal vesicles (ILVs), a complex called multivesicular bodies (MVBs), or late endosomes. If MVBs are not deconstructed, they merge to the plasma membrane to be released in the extracellular space as exosomes. Interestingly, studies performed in differentiated myocytes suggest that skeletal muscle may be able to facilitate cell-to-cell signaling through exosomes independently of MVBs, but by the direct release of exosomes through the plasma membrane [137].

Additionally, miRNAs consist of another regulatory component subjected to trafficking and that can potentially be myocyte-associated. Actually, Forterre et al. identified 180 miRNAs that were released by myoblast-specific exosomes in the conditioned media of differentiated C2C12 myoblasts, affecting major aspects of cellular homeostasis and function, such as proliferation, differentiation, survival, and regeneration [138].

Nevertheless, it should be mentioned that transporting systems involved in cell-to-cell-communication implicate a large spectrum of mechanisms that enable signal transduction by relocating a variety of molecules such as myokines and miRNAs. Those systems are not well described in skeletal muscle cells, and thus their detailed characterization is of great importance, as they may potentially mediate a substantial part of the exercise-induced, muscle-dependent regulation of the cancer microenvironment [139].

## 5. Conclusions and Future Perspectives

It is well established that physical inactivity is linked to high cancer incidence, while, conversely, regular exercise has been associated with decreased cancer risk and the regulation of cancer development and progression. Thus, there has been an increasing body of research focusing on the mechanistic interpretation of the anticancer effects of physical exercise and particularly the characterization of the molecular mechanisms that link exercise to tumor prevention and treatment. In this context, muscle-derived factors, myokines and miRNAs, secreted in response to contraction, appear to mediate exercise-induced beneficial effects and be responsible for inter-tissue communications that can control cancer dynamics. In the intercellular transport and delivery of muscle-secreted biomolecules, exosomes play an important role, delivering their content (miRNAs and myokines) into the target cells. The muscle secretome can modulate cancer evolution directly by affecting cancer cells and indirectly by stimulating the immune response and by compensating cancer-related sarcopenia, which affects patients’ quality of life.

Nevertheless, as research in the field of exercise oncology is growing, more factors, secreted by skeletal muscle cells in response to exercise and mediating its beneficial impact on cancer patients, are expected to be identified. In this context and since most of the research evidence comes from studies conducted in animal or cell culture models, further clinical research is warranted, focusing on the individualized optimization of the exercise protocol(s) depending on the disease characteristics and the responsiveness of each patient with cancer. Indeed, given the dose-dependent effect of physical activity on cancer progression and mortality, it remains a challenge to identify the particular characteristics of exercise protocols that can trigger optimal, long-term muscle adaptations against tumor development and cancer-associated sarcopenia. Moreover, the limitations of the animal and in vitro models, in terms of lacking a personalized and precision medicine approach, could be overcome and these research tools improved to better model human exercise and cancer progression. More specifically, combining human and cell culture studies might importantly contribute to the characterization of the exercise-induced secreted factors that potentially mediate the anti-cancer effects, e.g., by using the patients’ serum post exercise to treat cancer cells along with/or using inhibitors of specific myokine pathways. Furthermore, the utilization of in vitro exercise-mimetics, or models replicating skeletal muscle-specific aspects of exercise in vitro [140] could provide valuable insights in the mechanistic research of the link between exercise and the modulation of the tumor microenvironment. In addition, future research could employ muscle-specific conditional knockout of key myokine(s) in animal tumor models to map out the role of muscle-derived factors in the inter-tissue communication and the anticancer effects triggered by exercise.
